# Thermally induced deterioration behaviour of two dolomitic marbles under heating–cooling cycles

**DOI:** 10.1098/rsos.180779

**Published:** 2018-10-31

**Authors:** Zhong-jian Zhang, Jian-bin Liu, Biao Li, Xi-guang Yang

**Affiliations:** 1 Department of Civil Engineering, School of Engineering and Technology, China University of Geosciences, Beijing, no. 29 Xueyuan Road, Haidian District, Beijing 100083, People's Republic of China; 2 Department of Building, Civil & Environmental Engineering, Concordia University, 1455 de Maisonneuve Blvd, W., EV6.166 Montreal, Quebec, Canada H3G 1M8

**Keywords:** dolomitic marble, deterioration behaviour, large heating–cooling cycles, sonic wave velocity, uniaxial compressive strength

## Abstract

Thermally induced deterioration behaviour can cause severe weathering in marbles. Most previous studies focus on the deterioration behaviour of calcitic marbles. Relevant studies of dolomitic marbles are generally carried out under a ‘high temperature and low cycling times' condition. Little attention is focused on the deterioration behaviour in dolomitic marbles when they are subjected to a large quantity of heating–cooling cycles under a ‘low temperature and high cycling times’ condition. This paper presents experimental investigations on the thermally induced deterioration behaviour of two Beijing dolomitic marbles (Qingbaishi Marble (QM) and Hanbaiyu Marble (HM)) under heating–cooling cycles up to 1000 cycling times. The applied temperature range is from –20°C to 60°C which is to simulate the seasonal temperature variations in Beijing city, China. Related properties such as weight loss, three-dimensional microtopography, elastic wave velocity and uniaxial compressive strength were measured at certain cycles. The results indicate that thermally induced deterioration behaviour will result in a continuous weight loss in dolomitic marble samples. Mechanical properties of those two marbles are strongly affected by heating and cooling treatments, which were reflected by the reductions of dynamic Young's modulus and uniaxial compressive strength with an increase of thermal cycles. Compared with QM, HM displays a higher level of thermally induced deterioration which should be due to the abundance of quartz mineral.

## 1. Introduction

**Figure 1.**
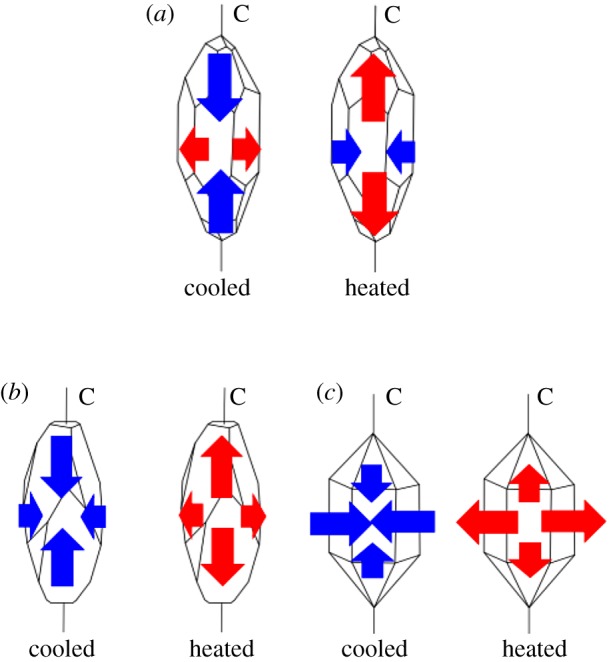
Sketch showing the anisotropic thermally induced deformation behaviours of (*a*) calcite, (*b*) dolomite and (*c*) quartz crystals. Under heating, the calcite crystal will expand along its *c*-axis while contracting in the direction perpendicular to the *c*-axis. Dolomite and quartz crystals will expand in directions parallel and perpendicular to *c*-axes. Modified after Steiger *et al*. [7].

Marbles have been widely used as building materials since ancient times due to their aesthetic value, workability and mechanical properties. They are the major components of many historic relics such as the Forbidden City and the Temple of Heaven in Beijing, China, the Parthenon Temple in Greece and the Taj Mahal in India. However, the structural integrity and appearance of those historic relics have increasingly been jeopardized by marble weathering [1,2]. It has been widely accepted that thermally induced deterioration behaviour is one of the major causes which are responsible for marble weathering [3–6]. Most previous studies focus on the thermally induced deterioration behaviour of calcitic marbles because of the strong anisotropic thermal strain behaviour of calcite mineral [1–4]. Shown in figure 1*a*, the calcite crystal has a positive linear thermal expansion coefficient (about 25.1 × 10^−6^ K^−1^) along the direction of the principal axis (*c*-axis); whereas it has a negative linear thermal expansion coefficient (about −5.6 × 10^−6^ K^−1^) along the direction perpendicular to the *c*-axis [7] (table 1). The differential expansion along different axes of calcite crystals during heating and cooling can generate high tensile stresses along crystal boundaries. Consequently, a progressive loss of the cohesion along grain boundaries may lead to the detachment of the grains or the disintegration of the structure [3,8,9]. Luque *et al*. [10] determined the durability of White Macael marble samples when they were subjected to changes in thermal conditions. They concluded that the progressive loss of cohesion along grain boundaries and an increase in porosity are starting points for marble degradation and facilitate the development of other pathologies. According to Leiss & Weiss [9], thermal cycling will result in significant residual strains in calcitic marbles, which is also treated as a major mechanism for progressive loss of cohesion among mineral grains.

**Table 1.** Linear thermal expansion coefficients for some common minerals present in marbles [7].

mineral	linear thermal expansion coefficient (K^−1^)		temp. range (°C)
	parallel to *c*-axis	perpendicular to *c*-axis	
calcite	25.1 × 10^−6^	−5.6 × 10^−6^	0–85
dolomite	25.8 × 10^−6^	6.2 × 10^−6^	24–700
quartz	7.7 × 10^−6^	13.3 × 10^−6^	0–80

**Figure 2.**
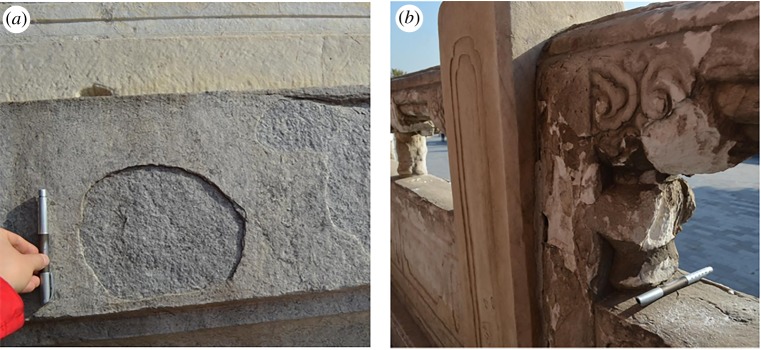
Severely weathered historic relics made of Beijing marbles: (*a*) contour scaling on a baluster base (made of Qingbaishi Marble (QM)) in the Temple of Heaven, Beijing, China, (*b*) granular disintegration on a baluster (made of Hanbaiyu Marble (HM)) in the Temple of Heaven, Beijing, China.

Compared with calcite, dolomite has a relatively lower level of anisotropy in the thermal expansion coefficient. The linear thermal expansion coefficients of dolomite mineral are 25.8 × 10^−6^ K^−1^ and 6.2 × 10^−6^ K^−1^ in directions parallel and normal to *c*-axis, respectively [11]. In figure 1*b*, the expansion property of dolomite crystal makes dolomitic marble more resistant to thermal deterioration when compared with that of calcitic marble. The studies on thermally induced damage in dolomitic marble are mainly at very high temperatures (up to 600°C) [12]. Few studies focused on the thermally induced deterioration behaviour of dolomitic marbles at temperatures comparable to the natural environment. Nevertheless, dolomitic marbles are the common construction materials of major historic relics such as the Temple of Heaven, in Beijing, China. Our recent on-site investigation at the Temple of Heaven, in Beijing, showed that some structures made of dolomitic marble were severely weathered (figure 2). Whether such severe weathering is related to the thermally induced deterioration behaviour needs to be investigated. In the context of climate change, our building structures are experiencing severe seasonable or daily temperature variations. For instance, temperatures of a building envelope measured during a hot summer in Beijing can be up to 55°C due to the sunlight, but the temperature can drop to −15°C in a cold winter. Thermal cycling may result in damage in marbles which will lead to strength degradation. The study on the physics of thermally induced deterioration behaviour in marbles under heating–cooling cycles is the fundamental for evaluating, preserving and restoring marble historic relics. Previous studies have limited experimental data on thermally induced deterioration behaviour of dolomitic marbles under natural thermal conditions. In addition, samples were not subjected to a large quantity of heating–cooling cycles. There is a gap to understand the thermally induced deterioration behaviour in marbles when they are subject to a large quantity of heating–cooling cycles.

In this research, we investigated the thermally induced deterioration behaviour of two dolomitic marbles under temperatures that simulate the natural thermal environment in Beijing, China. Samples were subjected to heating–cooling cycles up to 1000 times and relevant physical and mechanical properties were measured.

## 2. Materials used

Two kinds of dolomitic marble named Qingbaishi Marble (QM) and Hanbaiyu Marble (HM) were used in this study. Rock samples were quarried from Dashiwo town of FangShan district of Beijing. According to Zhang *et al*. [13], the historical records and the content of trace elements measured by X-ray fluorescence (XRF) indicate that those marbles used in the major historic relics in Beijing area such as the Temple of Heaven (figure 2) were quarried from the same site.

**Figure 3.**
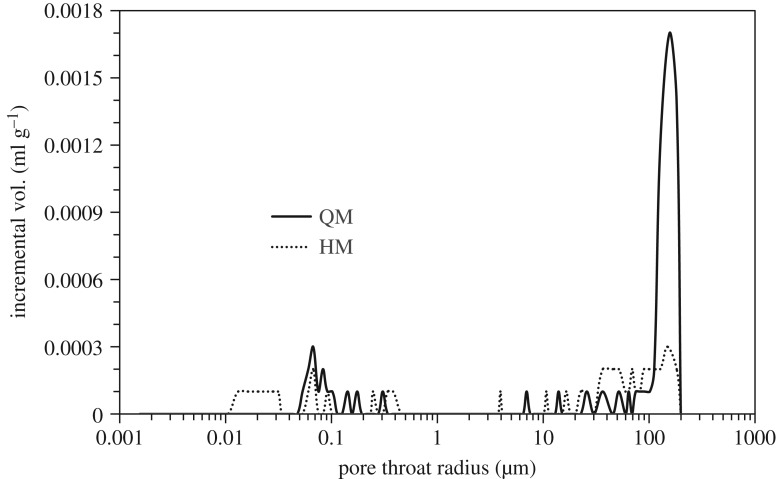
Pore size distributions of QM and HM.

**Figure 4.**
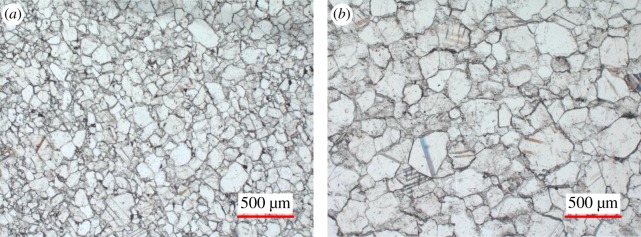
Photomicrographs of two freshly quarried Beijing marbles in plane-polarized light: (*a*) QM and (*b*) HM.

**Figure 5.**
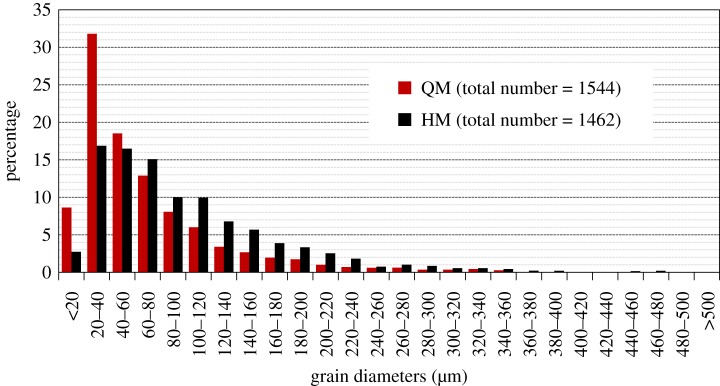
Grain size distributions of freshly quarried QM and HM.

As is presented in table 2, those two marbles have comparable values of bulk density (2.83 g cm^−3^). The uniaxial compressive strengths (UCS) of the undamaged marbles were determined before heating–cooling treatment. The porosities of those two marbles were also measured using the mercury intrusion porosimetry approach and are included in table 2. The pore throat size distribution curves are displayed in figure 3. It can be found that QM has more large pores when compared with HM. Mineral composition of marbles quarried and from architecture heritage sites were investigated using X-ray diffraction (XRD) analysis. Photomicrographs of thin sections under plane-polarized light of those two marbles are shown in figure 4. Image analysis was performed on those photomicrographs using Image-Pro Plus. The lithological characteristics of those two marbles were derived based on combined results from XRD analysis and optical analyses of thin slices. The results indicate that QM is mostly composed of dolomite (99%) (figure 4*a*). The impurities of mica and carbonaceous component are less than 1% (table 2). The average grain size of the minerals of QM is 67.4 µm. HM is mainly composed of dolomite (67%) and quartz (30%), as well as small quantities (3% in total) of feldspar and brown mica (figure 4*b*). The grain sizes of dolomite and quartz are between 20 and 600 µm, and between 100 and 800 µm, respectively. The average grain size of the minerals of HM is 97.9 µm. The detailed grain size distributions of QM and HM are shown in figure 5.

**Table 2.** Basic physico-mechanical properties and XRD results of freshly quarried QM and HM samples, as well as samples collected from heritage sites in Beijing, China. Note: QM-F and HM-F stand for freshly quarried QM and HM samples; QM-1, QM-2, HM-1, HM-2 stand for samples collected from historic relics.

name	bulk density (g·cm^−3^)	porosity	uniaxial compressive strength (MPa)	minerals and percentages (wt%)					
				no.	quartz	K-feldspar	dolomite	barite	mica
QM	2.824	0.019	146.89	QM-F	0.3	—	99.7	—	—
				QM-1	0.7	—	97.1	1.2	1.0
				QM-2	0.5	—	99.1	—	0.4
HM	2.878	0.013	115.50	HM-F	30.6	1.3	66.9	—	1.2
				HM-1	14.4	1.5	83.1	—	1.0
				HM-2	8.6	0.3	87.5	2.6	1.0

## 3. Methodology

Samples used to undergo thermal treatment were drilled, cut and classified into two groups (A and B) for different experimental purposes (table 3). Samples from group A were used for non-destructive tests such as weight measurement, microtopography (MT) and sonic wave velocity. Samples from group B were used to measure the uniaxial compressive strength. All the samples were subjected to heating–cooling cycles up to 1000 times. Related physical properties of weight loss, three-dimensional MT, elastic wave velocity and uniaxial compressive strength were obtained at certain cycles.

**Table 3.** Marble sample sizes in different groups for different experimental tests. Note: H, height, D, diameter.

group	shape	dimension (cm)	number of QM	number of HM	experiment tests
A	cube	5 × 5 × 5	10	10	mass remain ratio (weight loss), MT, sonic wave velocity
B	cylinder	H × D, 5 × 5	18	18	uniaxial compressive strength (UCS)

The heating–cooling treatment was carried out in the temperature range from −20°C to +60°C to simulate natural thermal conditions in the Beijing area. All of the samples were cleaned and dried at a temperature of 50°C for 24 h before thermal cycling processes. During the tests, all the samples were placed into the environmental chamber and subjected to heating and cooling cycles. The relative humidity in the chamber was in the range of 10–15%. A heating rate of 2°C min^−1^ and a cooling rate of 1°C min^−1^ were provided, to ensure the thermal equilibration of samples [14]. The samples were heated from 20°C to 60°C and kept for 20 min, then cooled from 60°C to −20°C and kept for 20 min, then heated to 20°C to complete one cycle. A full cycle takes 160 min and a total of 1000 cycles were carried out in the experiment.

**Figure 6.**
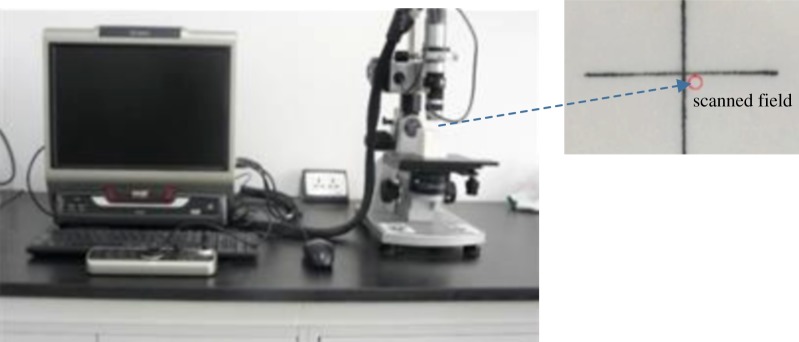
The applied digital microscope (Model VHX-2000) and the scanned field in a rock surface.

Samples from group A were taken out to measure the weight after every 100 cycles. The weight loss for each sample was derived accordingly. A digital microscope (model VHX-2000) was used to scan the marked surfaces (figure 6) of a QM sample and an HM sample at different thermal cycles. The three-dimensional MT of those two samples could be produced accordingly.

Samples from group A were also used to measure ultrasonic velocities every 100 cycles. Both P-wave (compressive wave) and S-wave (shear wave) velocities were measured using transducers with a frequency of 250 kHz. The data were collected using a Pundit PL-200 ultrasonic pulse velocity testing equipment.

Samples in group B have a height of 5 cm and a diameter of 5 cm. The reason for using a small sample size is to save the marble material and make sure that the environmental chamber has enough space to accommodate such a large quantity of samples. According to the International Society for Rock Mechanics suggested methods for uniaxial compression tests [15], the maximum ratio of the grain diameter to specimen diameter is smaller than 0.1. As is shown in figure 5, the grain sizes of those rock samples are less than 5 mm, which fulfils such requirement. Six samples from group B (three QM and three HM) were taken out after every 200 cycles to measure the uniaxial compressive strength (UCS). As the UCS test is a destructive test, samples after UCS tests will not be placed back into the environmental chamber. The tests were performed using a high stiffness compression loading frame (Model 50-C43Z00). A loading rate of 0.5 MPa s^−1^ was applied for all the tests and the peak strength data were recorded. During those tests, our displacement monitoring transducers were not in good function; thus, we are not able to obtain the parameters of static Young's modulus and Poisson's ratio.

## 4. Results

### 4.1. Weight loss and three-dimensional microtopography

**Figure 7.**
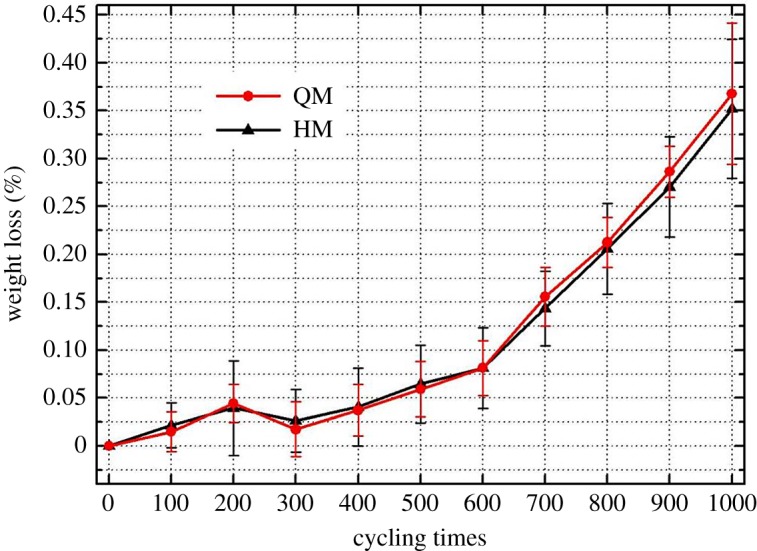
Weight loss (%) of samples after every 100 heating–cooling cycles.

The weight loss for QM and HM samples were derived and are plotted in figure 7. Each point accompanied with the standard variation corresponds to an average value of 10 measurements. Generally, both samples lose weight with the increase of thermal cycles. When the thermal cycles approach a value of 600, both samples start to have a major weight loss. After the tests, we were able to find white rock flour by touching samples' surfaces. The difference in the weight loss values of QM and HM is negligible.

In the applied temperature range, there is little opportunity to cause gasification and evaporation of carbonaceous component in those samples. The particle detachment is the major reason for samples' weight loss. Anisotropic expansion and contraction of dolomite crystals and quartz crystals during thermal processes will cause microcracks in dolomitic marbles. Thermally induced microcracks will firstly be generated along a sample's surface, because the exposed sample surface is under an unconfined stress state and it is also firstly subjected to a thermal gradient. The generation of microcracks is accompanied by losing very fine particles at lower thermal cycles. At higher thermal cycles, microcracks accumulate to such an extent that grain particles on the free surface start to detach from the main body and a new surface is created. This explains the transition behaviour at a thermal cycle of 600 times observed in figure 7. It can be estimated that the weights of those samples will continue to decrease at thermal cycles higher than 1000 times.

**Figure 8.**
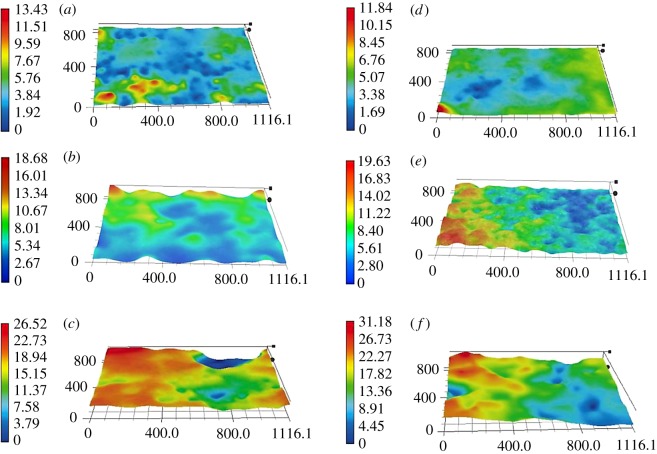
MT of sample surfaces at a marked position in the initial condition and after 600 and 1000 thermal cycles. (*a*) QM in the initial condition; (*b*) QM after 600 thermal cycles; (*c*) QM after 1000 thermal cycles; (*d*) HM in the initial condition; (*e*) HM after 600 thermal cycles; (*f*) HM after 1000 thermal cycles.

The three-dimensional microtopographic images of the surfaces of those two dolomitic marbles were obtained and are shown in figure 8. After 1000 thermal cycles, surfaces of both samples have significant undulation. Compared with QM, HM has a rougher surface figure 8.

As is presented in table 2, QM is mostly composed of dolomite. However, HM has a considerable amount of quartz. The quartz crystal has a larger linear thermal expansion coefficient along the direction perpendicular to the *c*-axis than that of the direction parallel with the *c*-axis (figure 1*c* and table 1). The anisotropic thermal expansive behaviour of quartz crystal is very different from that of dolomite crystal, which will increase the chance of bringing thermally induced irreversible strains. Thereby, it is expected that HM is more easily involved in thermally induced deterioration behaviour when compared with QM.

### 4.2. Sonic wave velocity

**Figure 9.**
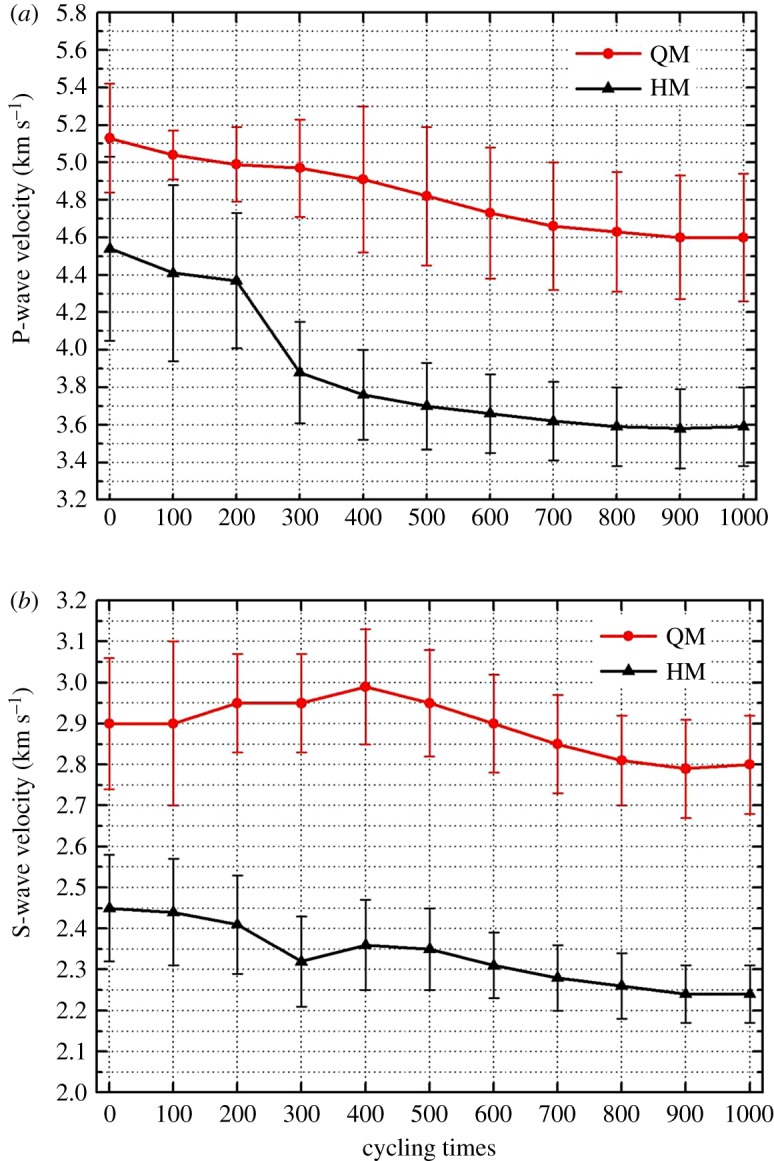
P-wave velocities (*a*) and S-wave velocities (*b*) of marble samples after every 100 heating–cooling cycles.

Results of the P-wave and S-wave velocities of samples after every 100 heating–cooling cycles are presented in figure 9. Each point also represents an average value of 10 measurements and the standard variation is provided. In general, QM has a higher sonic wave velocity than that of HM. Both marbles display a decreasing trend in sonic wave velocities with an increase of thermal cycles. However, the decreasing trends for those two marbles are different. Shown in figure 9*a*, the P-wave velocity of QM continuously decreases with the increase of thermal cycles and the total reduction is in an amount of 10.3% at a thermal cycle of 1000 times. By contrast, the P-wave velocity of HM has a major reduction (drop by 15.1%) in the first 300 cycles. The S-wave velocities of both samples are also sensitive to the thermal cycles (figure 9*b*). The S-wave velocity of QM displays an increasing trend in the first 400 cycles followed by a continuous decreasing trend latterly. Such a fluctuation behaviour is not observed in the S-wave velocity of HM. The difference should be due to their mineralogy difference.

Based on measured results of P-wave and S-wave velocities of those samples, the dynamic Young's modulus and dynamic Poisson's ratio, can be calculated using the following equation [16].

**Figure 10.**
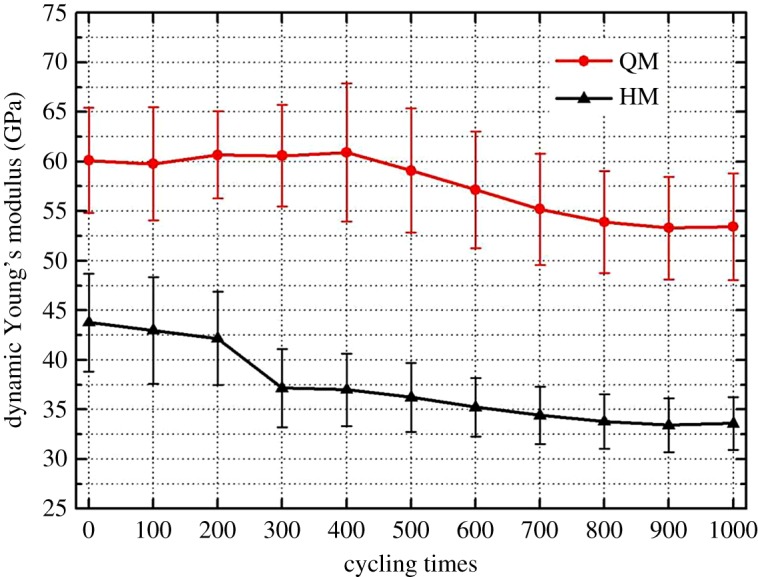
Dynamic Young's modulus of samples after every 100 heating–cooling cycles.

**Figure 11.**
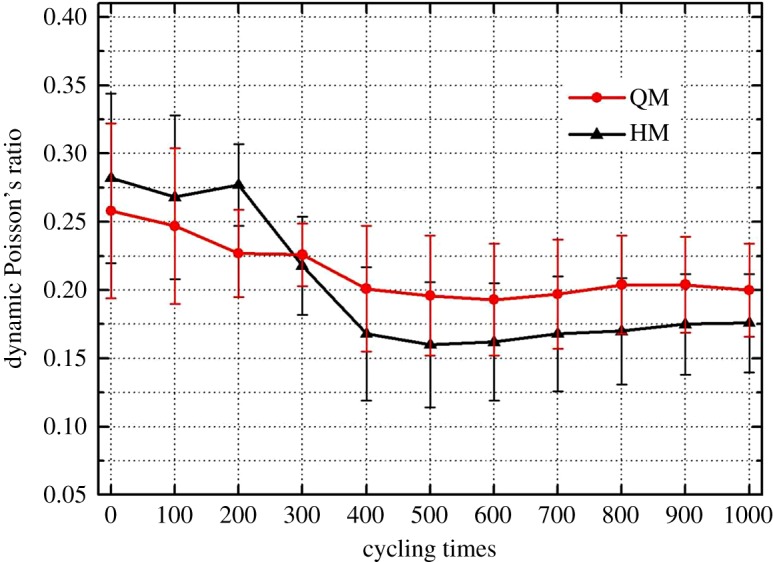
Dynamic Poisson's ratio of samples after every 100 heating–cooling cycles.

4.1 $$\begin{array}{rl} E_{d} & =\rho_{b}\frac{3V_{p}^{2}-4V_{s}^{2}}{V_{p}^{2}-V_{s}^{2}}V_{s}^{2}\\ \mathrm{and}\;\;\;\;\;\;\;\;\nu_{d} & =\frac{1}{2}\frac{{\left(V_{p}/V_{s}\right)}^{2}-2}{{\left(V_{p}/V_{s}\right)}^{2}-1}, \end{array}\}$$

where *V_p_* is P-wave velocity (km s^−1^), *V_s_* is S-wave velocity (km s^−1^) and *ρ*_*b*_ is the density of samples (kg m^−3^). Dynamic Young's modulus results of those samples at thermal cycles are shown in figure 10. Generally, the thermally induced deterioration behaviour of those two dolomitic marbles can be reflected by the reduction of stiffness with an increase of thermal cycles. Compared with QM, HM displays a higher level of deterioration, which should be due to the abundance of quartz mineral. The fluctuation in the curve of QM is due to the similar trend observed in that of the S-wave velocity. The anisotropic thermal expansion behaviour of dolomite crystal should be the major reason for having such variations. Further studies on the thermal cycling effect on the anisotropic sonic wave velocity properties of marbles are recommended. The dynamic Poisson's ratio results of those samples at thermal cycles are displayed in figure 11, which also indicates that Poisson's ratio of HM (drop by 37.5%) is more sensitive to the thermal cycles when compared with that of QM (drop by 22.3%).

As sonic wave velocities are closely related to the microstructure of a brittle rock [17,18]. The significant drop in sonic wave velocities of those marbles indicates an extension or opening of microcracks among mineral grain boundaries. The increased pore space corresponds to the initial stage of marble degradation, which will facilitate the physical and chemical weathering processes.

### 4.3. Uniaxial compressive strength

**Figure 12.**
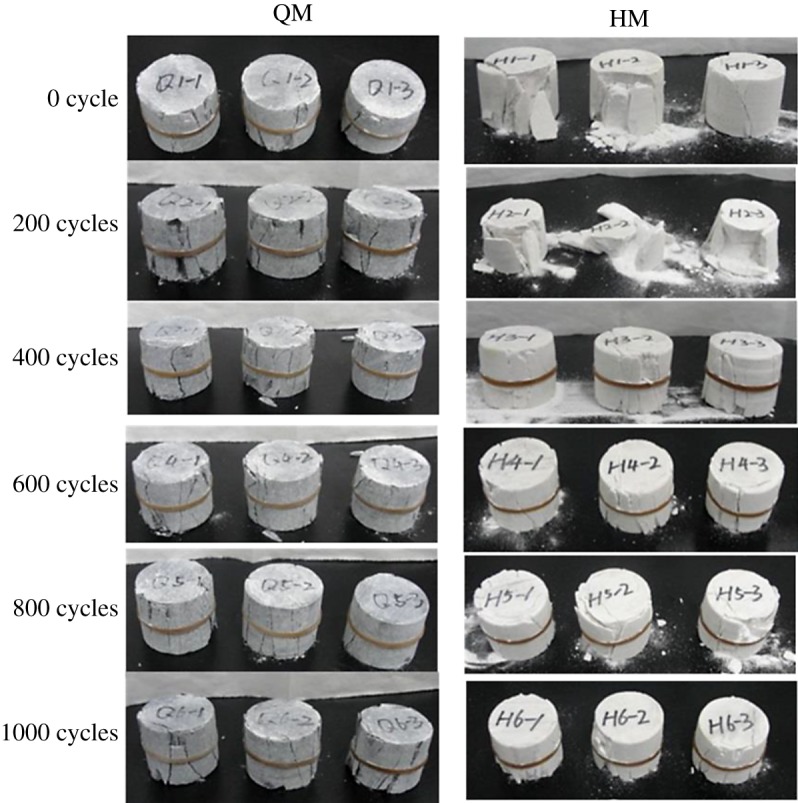
Pictures showing the failed marble samples after uniaxial compressive tests.

**Figure 13.**
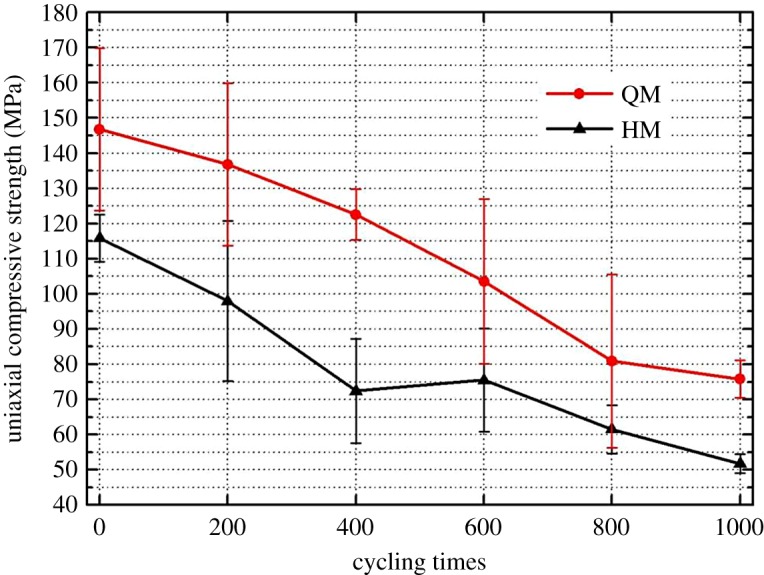
UCS of samples after every 200 heating–cooling cycles.

Both QM and HM display a brittle failure mode in the uniaxial compressive tests (figure 12). Each point represents an average value of three measurements. Compared with QM, HM samples tend to have more powder trash at failure due to mineral grain crushing. The reason could be due to the existence of quartz mineral and the larger grain particle size. The uniaxial compressive strength data of those samples were plotted against the thermal cycles and shown in figure 13. There is a continuous strength degradation trend for both QM and HM samples, which implies that thermally induced deterioration behaviour in dolomitic marbles has an accumulative effect. Eventually, samples tend to completely lose the compressive strength. In addition, the uniaxial compressive strength of HM has a major drop in strength in the first 400 cycles, which was also observed in the curve of P-wave velocity. After 1000 thermal cycles, the UCS of QM and HM reduced by 45% and 52%, respectively. The inclusion of quartz mineral in dolomitic marble should have promoted the thermally induced deterioration behaviour in HM samples.

## 5. Concluding remarks

This study presents the experimental investigations on the thermally induced deterioration behaviour of two Beijing dolomitic marbles (QM and HM) under heating–cooling cycles. The applied thermal cycling is up to 1000 cycles in a temperature range from −20°C to 60°C. Several conclusions are addressed as the following:

— When Beijing dolomitic marbles are subjected to a large quantity of heating–cooling cycles, the thermally induced deterioration behaviour can be significant even if they are only under a varied temperature condition which is comparable to that of the natural environment.
— Thermally induced deterioration behaviour will result in a continuous weight loss in marble samples with the increase of thermal cycles. The weight loss can also be viewed by the generated undulations on samples' surfaces using a digital microscope.
— Mechanical properties of those two marbles are strongly affected by heating and cooling treatments, which were reflected by the reductions of dynamic Young's modulus and uniaxial compressive strength with an increase of thermal cycles. Samples tend to completely lose the compressive strength when they are continuously subjected to heating–cooling cycles. Compared with QM, HM displays a higher level of thermally induced deterioration which should be due to the abundance of quartz mineral.

## Data accessibility

All data and materials are included in the manuscript.

## References

[1] Gauri KL, Hagerty D, Ullrich CR 1972 Comparative physical properties of weathered impregnated and unimpregnated marble. Eng. Geol. 6, 235–250. (10.1016/0013-7952(72)90009-9)

[2] Siegesmund S, Ullemeyer K, Weiss T, Tschegg EK 2000 Physical weathering of marbles caused by anisotropic thermal expansion. Int. J. Earth Sci. 89, 170–182. (10.1007/s005310050324)

[3] Yavuz H, Demirdags S, Caran S 2010 Thermal effect on the physical properties of carbonate rocks. Int. J. Rock Mech. Min. Sci. 47, 94–103. (10.1016/j.ijrmms.2009.09.014)

[4] Rodríguez-Gordillo J, Sáez-Pérez MP 2006 Effects of thermal changes on Macael marble: experimental study. Constr. Build Mater. 20, 355–365. (10.1016/j.conbuildmat.2005.01.061)

[5] Kracke TS, Ruedrich J, Schwarzburg R 2010 Jewish cemetery in Hamburg Altona (Germany): State of marble deterioration and provenance. Eng. Geol. 115, 200–208. (10.1016/j.enggeo.2009.07.008)

[6] Siegesmund ST, Fuller ER 2003 Thermal degradation of marble: indications from finite-element modelling. Build Environ. 38, 1251–1260. (10.1016/S0360-1323(03)00082-9)

[7] Steiger M, Charola AE, Sterflinger K 2011 Weathering and deterioration. In Stone in architecture: properties, durability (eds Siegesmund S, Snethlage R), pp. 227–316. Berlin, Germany: Springer.

[8] Ferrero AM, Migliazza M, Spagnoli A, Zucali M 2014 Micromechanics of intergranular cracking due to anisotropic thermal expansion in calcite marbles. Eng. Fract. Mech. 130, 42–52. (10.1016/j.engfracmech.2014.01.004)

[9] Leiss B, Weiss T 2000 Fabric anisotropy and its influence on physical weathering of different types of Carrara marbles. J. Struct. Geol. 22, 1737–1745. (10.1016/S0191-8141(00)00080-8)

[10] Luque A, Cultrone G, Mosch S, Siegesmund S, Sebastian E, Leiss B 2010 Anisotropic behaviour of White Macael marble used in the Alhambra of Granada (Spain): the role of thermohydric expansion in stone durability. Eng. Geol. 115, 209–216. (10.1016/j.enggeo.2009.06.015)

[11] Reeder RJ, Markgraf SA 1986 High-temperature crystal chemistry of dolomite. Am. Mineral. 71, 795–804.

[12] Peng J, Rong G, Cai M, Yao M, Zhou C 2016 Comparison of mechanical properties of undamaged and thermal-damaged coarse marbles under triaxial compression. Int. J. Rock Mech. Min. Sci. 83, 135–139. (10.1016/j.ijrmms.2015.12.016)

[13] Zhang ZJ, Yang XG, Ye FJ, Zhou H, Zhang T 2015 Microscopic characteristics of petrography and discussion on weathering mechanism of Fangshan marble in Beijing. J. Eng. Geol. 23, 279–286. In Chinese.

[14] Cantisani E, Pecchioni E, Fratini F, Garzonio CA, Malesani P, Molli G 2009 Thermal stress in the Apuan marbles: relationship between microstructure and petrophysical characteristics. Int. J. Rock Mech. Min. Sci. 46, 128–137. (10.1016/j.ijrmms.2008.06.005)

[15] ISRM. 1979 Suggested methods for determining the uniaxial compressive strength and deformability of rock materials. Int. J. Rock Mech. Min. Sci. 16, 135–140.

[16] Mavko G, Dvorkin J, Mukerji T 2009 The rock physics handbook. Cambridge, UK: Cambridge University Press.

[17] Tomás RV, Ivorra S, Grediaga A 2014 Relationship between static and dynamic elastic modulus of calcarenite heated at different temperatures: the San Julián's stone. Bull. Eng. Geol. Environ. 73, 1–9. (10.1007/s10064-013-0486-3)

[18] Nara YA, Kubo T 2016 P-wave propagation in dry rocks under controlled temperature and humidity. Int. J. Rock Mech. Min. Sci. 86, 157–165. (10.1016/j.ijrmms.2016.04.011)

